# Epigenetic population differentiation in field‐ and common garden‐grown *Scabiosa columbaria* plants

**DOI:** 10.1002/ece3.3931

**Published:** 2018-02-25

**Authors:** Maartje P. Groot, Niels Wagemaker, N. Joop Ouborg, Koen J. F. Verhoeven, Philippine Vergeer

**Affiliations:** ^1^ Experimental Plant Ecology Institute for Water and Wetland Research Radboud University Nijmegen Nijmegen The Netherlands; ^2^ Department of Terrestrial Ecology Netherlands Institute of Ecology (NIOO‐KNAW) Wageningen The Netherlands; ^3^ Plant Ecology and Nature Conservation Group Wageningen The Netherlands

**Keywords:** AFLP, common garden, DNA methylation, epigenetic memory, MS‐AFLP, population epigenetics

## Abstract

Populations often differ in phenotype and these differences can be caused by adaptation by natural selection, random neutral processes, and environmental responses. The most straightforward way to divide mechanisms that influence phenotypic variation is heritable variation and environmental‐induced variation (e.g., plasticity). While genetic variation is responsible for most heritable phenotypic variation, part of this is also caused by nongenetic inheritance. Epigenetic processes may be one of the underlying mechanisms of plasticity and nongenetic inheritance and can therefore possibly contribute to heritable differences through drift and selection. Epigenetic variation may be influenced directly by the environment, and part of this variation can be transmitted to next generations. Field screenings combined with common garden experiments will add valuable insights into epigenetic differentiation, epigenetic memory and can help to reveal part of the relative importance of epigenetics in explaining trait variation. We explored both genetic and epigenetic diversity, structure and differentiation in the field and a common garden for five British and five French *Scabiosa columbaria* populations. Genetic and epigenetic variation was subsequently correlated with trait variation. Populations showed significant epigenetic differentiation between populations and countries in the field, but also when grown in a common garden. By comparing the epigenetic variation between field and common garden‐grown plants, we showed that a considerable part of the epigenetic memory differed from the field‐grown plants and was presumably environmentally induced. The memory component can consist of heritable variation in methylation that is not sensitive to environments and possibly genetically based, or environmentally induced variation that is heritable, or a combination of both. Additionally, random epimutations might be responsible for some differences as well. By comparing epigenetic variation in both the field and common environment, our study provides useful insight into the environmental and genetic components of epigenetic variation.

## 1 INTRODUCTION

Plants often show differences in morphology and life history within and between populations. These differences arise because different environments lead to different selection pressures. Selection pressures shape adaptive genetic variation and, in combination with random processes such as drift, lead to heritable differences in plant phenotype. The phenotype of an individual is determined by the interactions between the environment and its genotype, which includes both evolutionary adaptation and plasticity (Pigliucci, 2005; Sultan, 2000). An underlying mechanism of plasticity and possibly adaptation that may additionally explain variation in morphology and life history are epigenetic processes (Bossdorf, Richards, & Pigliucci, 2008).

Epigenetic variation can influence gene expression without changes in the underlying DNA sequence and can therefore ultimately influence phenotype (Bossdorf, Arcuri, Richards, & Pigliucci, 2010; Bossdorf et al., 2008; Cortijo et al., 2014; Cubas, Vincent, & Coen, 1999; Johannes et al., 2009). Additionally, the environment can directly influence epigenetic variation (Bossdorf et al., 2008; Verhoeven, Jansen, van Dijk, & Biere, 2010). Recent studies have shown that epigenetic variation is relatively common in plants and that environmental‐induced epigenetic changes can in some cases be stably inherited to the following generations (Jablonka & Raz, 2009; Verhoeven et al., 2010). Epigenetic mechanisms include DNA methylation, histone modification, and small RNAs (Rapp & Wendel, 2005). DNA methylation is the most commonly studied epigenetic mechanism (Bossdorf et al., 2008; Schulz, Eckstein, & Durka, 2013).

Epigenetic mechanisms can be an important component of phenotypic variation when epigenetic variation operates, at least partly, autonomous from genetic variation because it can then explain variation that was not explained by the underlying genetic variation (Bossdorf et al., 2008). An additional interesting part of epigenetic mechanisms is that they may mediate responses to environmental changes that persist into offspring (transgenerational effects), extending the scope of phenotypic plasticity across generations.

There is an increasing number of studies exploring epigenetic variation in natural populations (Abratowska, Wąsowicz, Bednarek, Telka, & Wierzbicka, 2012; Avramidou, Ganopoulos, Doulis, Tsaftaris, & Aravanopoulos, 2015; Foust et al., 2016; Herrera & Bazaga, 2010; Lira‐Medeiros et al., 2010; Ma, Song, Yang, Zhang, & Zhang, 2013; Nicotra et al., 2015; Preite et al., 2015; Richards, Schrey, & Pigliucci, 2012; Rico, Ogaya, Barbeta, & Peñuelas, 2014; Sáez‐Laguna et al., 2014; Schulz, Eckstein, & Durka, 2014; Wu et al., 2013; Yu et al., 2013). A number of these studies correlate epigenetic variation with phenotypic traits such as seed size variability and several whole plant, leaf and regenerative traits (Herrera, Medrano, & Bazaga, 2014; Medrano, Herrera, & Bazaga, 2014). Several studies report natural populations that are epigenetically differentiated (Avramidou et al., 2015; Herrera & Bazaga, 2010; Lira‐Medeiros et al., 2010; Ma et al., 2013; Preite et al., 2015; Richards et al., 2012; Sáez‐Laguna et al., 2014). Often such epigenetic differentiation is correlated with different habitats or environmental stresses and, at least to some extent, independent from the underlying genetic variation (Abratowska et al., 2012; Foust et al., 2016; Lira‐Medeiros et al., 2010; Ma et al., 2013; Preite et al., 2015; Sáez‐Laguna et al., 2014). Interestingly, in studies on offspring from natural populations, indications for the heritability of epigenetic differences were found (Preite et al., 2015; Richards et al., 2012; Schulz et al., 2014). To date, nearly all studies on these mechanisms are performed either in the field or in a common environment (and not in both, but see Nicotra et al., 2015).While the combination of screening population, epigenetic variation both in the field and in a common garden environment allows the differentiation between environment‐induced epigenetic variation and epigenetic memory.

Here, we used AFLP and MS‐AFLP techniques to study genetic and epigenetic variation within and between populations. MS‐AFLP is a suitable method to assess epigenetic differentiation in nonmodel plant populations and to uncover global correlations between genetic variation, epigenetic variation, habitats, and phenotype (Alonso, Pérez, Bazaga, Medrano, & Herrera, 2016; Schrey et al., 2013; Schulz et al., 2013). We sampled 10 different populations of *Scabiosa columbaria*, an outcrossing species with high genetic variation within populations and phenotypic differentiation among populations (Pluess & Stöcklin, 2004; Waldmann & Andersson, 1998). Plants from these populations were individually sampled and measured. In addition, seedlings from these populations were grown in a common garden and sampled and measured to study the extent of transmittance of epigenetic population differentiation in this generation. We compared Q_ST_ to ɸ_ST_ to help to distinguish if differentiation between populations is the result of natural selection or neutral random processes such as drift (Merilä & Crnokrak, 2001; Scheepens, Stöcklin, & Pluess, 2010; Whitlock, 2008).

The different populations and countries were chosen to study the genetic differentiation in combination with geographic distance, and the epigenetic variation in relation with geographic and climatic differences. We asked the following questions: (i) Are populations epigenetically differentiated? (ii) Is epigenetic variation correlated with phenotypic variation and is epigenetic variation independent of genetic variation? (iii) Can we detect evidence for epigenetic memory?

## 2 MATERIAL AND METHODS

### 2.1 Study species

*Scabiosa columbaria* L. is a short‐lived perennial herb that occurs on dry, calcareous grasslands in Europe. It is a protandrous, insect pollinated, mainly outcrossing species, although it is self‐compatible. *Scabiosa columbaria* grows a basal rosette and flowers from June till September with branded stalks with several flowering heads. Each flower head has around 50–70 florets that, when successfully fertilized, produce a single‐seeded fruit (Ouborg, Van Treuren, & Van Damme, 1991; Picó, Ouborg, & Van Groenendael, 2004; Van Treuren, Bijlsma, Ouborg, & Van Delden, 1993). In 2009, seeds and leaf material were collected from 20 individuals per population from five British (UK) and five French (FR) populations (Table 1). Only large populations (>500 individuals) were selected. The main differences between the sites are given in Table 1. The locations of the populations were chosen along its western European North–South distribution range. Hence, the range covers a large environmental gradient with large climatological differences between populations. Seeds were stored in paper bags at room temperature until used for germination. Leaf material, collected only from fresh and undamaged leaves, was immediately dried in silica gel and upon arrival in the lab all leaf material was stored at −80°C to minimize risk of epigenetic changes during storage.

**Table 1:** Site properties of sampled *Scabiosa columbaria* populations including total genetic and epigenetic diversity

Site	Latitude	Longitude	Altitude (m asl)	Population size	AFLP				MS‐AFLP Field				MS‐AFLP Common garden			
					No. of samples	No. of bands	*P* (%)	*H*	No. of samples	No. of bands	*P*_epi_ (%)	*H*_epi_	No. of samples	No. of bands	*P*_epi_ (%)	*H*_epi_
FR 1	45.58356	2.93492	980	5,000	9	141	75.0	0.574	7	16	53.9	0.467	5	13	52.9	0.426
FR 2	45.34833	0.53625	147	1,000	10	142	82.6	0.635	9	20	73.1	0.559	8	12	70.6	0.597
FR 3	45.49343	−0.80086	30	500–1,000	9	135	71.5	0.523	8	20	65.4	0.494	9	15	88.2	0.607
FR 4	49.63469	1.33933	148	>1,0000	9	143	75.0	0.572	10	21	69.2	0.543	16	14	76.5	0.600
FR 5	50.79822	1.9455	100	1,000–5,000	10	143	79.9	0.595	8	18	57.7	0.524	10	14	76.5	0.549
UK 6	50.897639	−0.056528	170	>10,000	9	141	72.9	0.549	8	20	73.1	0.564	10	13	76.5	0.565
UK 7	50.886222	−0.831919	60	5,000	10	142	76.4	0.566	8	18	57.7	0.430	9	13	76.5	0.675
UK 8	51.366333	−1.841694	200	1,000	9	140	77.8	0.604	8	21	69.2	0.568	6	11	64.7	0.572
UK 9	53.270056	−1.740472	240	5,000–10,000	5	139	64.6	0.535	9	15	50.0	0.364	5	11	52.9	0.470
UK 10	54.688111	−1.514556	115	5,000	8	140	72.2	0.548	6	23	73.1	0.622	10	12	58.8	0.402

*P* (%) is the percentage of polymorphic bands. *H* is Shannon's information index based on the genetic loci. *P*_epi_ is the percentage of polymorphic epiloci and *H*_epi_ is Shannon's information index based on epiloci.

### 2.2 Common garden experiment

For the common garden experiment, we used the seeds collected in each of the five UK and five FR populations. All seeds were stored similarly and most mother plants produced seedlings. The average germination percentage per mother plant was 64% (ranging from 50% to 85%). From our experience, these are normal germination rates for natural populations of *S. columbaria* using fresh seed material. Of each mother plant, all available seeds were used for germination. Seeds were placed in a petri dishes with filter paper, which was moistened with deionized water. Germinating seeds were kept in a climate chamber with a 20°C/16°C (day/night) temperature regime, long day (16 hr/8 hr, day/night), and light conditions of 236 μmol m^−2^ s^−1^. After germination, five seedlings per mother plant were individually planted in peat Jiffypots^®^ (6 cm diameter, Jiffy Products International BV, Moerdijk, the Netherlands) filled with soil from the common garden field site. The individual pots were subsequently placed in an unheated greenhouse, where they stayed for 12 weeks. At the end of May 2013, when ground temperatures were no longer expected to drop below 0°C, all plants were planted in a randomized block design (with five blocks and a single replicate for each mother per block) in an open common garden field site at the experimental garden of Radboud University, Nijmegen, the Netherlands. Individual plants were placed at 25 cm intervals with four plants per row.

### 2.3 Phenotypic measurements

In both field and common garden environment, the biomass index [BMI; the product of the number of leaves and the length and width of the largest leaf; a nondestructive way to measure biomass (Vergeer, Wagemaker, & Ouborg, 2012)], number of flowering stems, number of flowers on each flowering stem, and the total number of flowers per plant was measured. The biomass index for the field plants was measured at time of seed set. For the garden‐grown plants, biomass index was measured when they were placed in the common garden (week 1) and approximately 2 weeks before bolting (after 11 weeks, beginning of August 2013). The data of the first measurement were used in the analysis of the second measurement data to correct for differences in initial biomass at time of planting. Additionally, in the common garden, we also determined bolting date and day of opening of the first flower (flowering time). After seed set (at the end of November 2013, before temperature dropped below zero and before plants had started to senescence), all plants were harvested. After 1‐week oven drying at 70°C, we measured reproductive biomass (inflorescence and flower mass), biomass of the plant excluding the reproductive biomass and by combining those total biomass. A Pearson's correlation test showed a strong correlation between total biomass and biomass index measured before bolting in the common garden (*r* = .69, *p*‐Value <.0001).

### 2.4 DNA isolation, AFLP, and MS‐AFLP

DNA was isolated from 10 individuals per population from the common garden‐grown plants for both AFLP and MS‐AFLP analysis. All selected plants came from different mother plants. In addition, DNA was isolated from 10 individuals per population from the field‐collected leaf material for MS‐AFLP analyses of field‐collected plants, which were not necessarily the same plants as the mother plants of which seeds were collected. DNA was isolated from approximately 1.5 cm^2^ leaf material using the Nucleo spin 8 plant II kit (Machery‐nagel, the Netherlands). DNA amount was quantified using Qubit^®^ 1.0 Fluorometer (ThermoFisher Scientific, the Netherlands). To test if the sample size of 10 individuals resulted in reliable estimations of the effects, coefficients of variance for BMI were calculated for each population with random selections of samples ranging from *n* = 3 to *n* = 20. These random selections show that generally a sample size of *n* = 10 has a minimal effect on the variance as compared to larger sample sets of *n* = 20 (Data S1).

For genotyping the five UK and five FR populations, the amplified fragment length polymorphism (AFLP) method was used, with EcoRI as a rare‐cutting enzyme and MseI as the frequent cutter (Vos et al., 1995). In order to analyze the epigenetic variation between populations and countries, we used an adaptation from the AFLP method, the methylation‐sensitive amplified fragment length polymorphism (MS‐AFLP) where the frequent cutter MseI is replaced in two parallel batches by two methylation‐sensitive cutters, MspI and HpaII, which cut the same 5′‐CCGG restriction site but differ in methylation sensitivity (Keyte, Percifield, Liu, & Wendel, 2006; Reyna‐Lopez, Simpson, & Ruiz‐Herrera, 1997).

See Table S1 for a complete overview of all adapters and primers used for both AFLP and MS‐AFLP protocols. The AFLP and MS‐AFLP protocols were adapted from Vergeer et al. (2012).

Field and common garden samples were analyzed separately and were randomized between plates, to prevent plate bias. In addition, all MS‐AFLP samples were run in duplo, for both MspI and HpaII.

We analyzed fragments of both AFLP and MS‐AFLP with GENEMARKER version 2.6.3 (Softgenetics) and scored fragments between 98 and 600 base pairs. Marker loci were scored when the peaks were at least three times higher than the noise, and when in the individual sample the peak height signal was above 100. Additionally, mismatching Duplo's were checked manually and were only included if both peaks showed a clear signal above 50, otherwise, they were excluded. Mismatched Duplos were generally <10% per plate. Samples that failed in one or more primer combinations were excluded from further analysis, just as loci with less than 5% variability for both AFLP and MS‐AFLP. This resulted in a total of 88 AFLP samples with 144 polymorphic loci, 88 MS‐AFLP samples from the common garden with 140 polymorphic loci and 81 MS‐AFLP samples from the field with 109 polymorphic loci. Fragments were scored as methylated (fragment present in EcoRI/MspI or EcoRI/HpaII, but not in both, fragment type II or III) or nonmethylated (fragment present in both EcoRI/MspI and EcoRI/HpaII, fragment type I). The absence of fragments was scored as missing data (fragment type IV) because in this case, it is not possible to distinguish between complete methylation and genetic restriction site polymorphism as the cause of fragment absence (Schulz et al., 2013; Vergeer et al., 2012).

### 2.5 Statistical analysis

In the phenotypic data of both the field and the common garden, we tested for differences between countries and between the populations within each country. We expect British populations to differentiate from French populations due to their geographic isolation. Differences between countries were analyzed using linear mixed effect models with country as a fixed effect and population nested within country as random effect (Bates, Maechler, Bolker, & Walker, 2014). For the common garden, data block was included as a random factor. The denominator degrees of freedom and *p*‐Values for the linear mixed effects models were calculated using the lmerTest R package (Kuznetsova, Brockhoff, & Christensen, 2015). In addition, we tested for population effects within country as the populations were selected along an environmental and climatic gradient, also within countries. For this analysis, these data were split in a French and British dataset to test for a population effect. Then, we performed a second model, separately for FR and UK, with populations as the fixed effect. Tukey's post hocs were performed to test whether there were phenotypic differences between populations. For the field data, we performed linear mixed effect models, using generalized least squares (Pinheiro, Bates, DebRoy, & Sarkar, 2015). For the common garden, linear mixed effect models were fitted to be able to include block as a random factor (Pinheiro et al., 2015). All models were adjusted for variance heterogeneity leading to a better model fit (using the *varIden* function in the R package nlme). The percentage of variance explained by country and population (population variance was calculated separately for FR and UK populations) was estimated using the *lmer* function from the lme4 package, for all phenotypic traits (Bates et al., 2014).

Q_ST_, the genetic divergence in functional quantitative traits (Spitze, 1993), was calculated for all traits shared between Field and Common garden (biomass index, inflorescence height, number of inflorescences and number of flowers), using the variances calculated with the linear mixed models described above (Steinger, Haldimann, Leiss, & Müller‐Schärer, 2002; Whitlock, 2008).

The binary AFLP and MS‐AFLP data were analyzed using a band‐based strategy, where the presence or absence band pattern was compared between samples (Bonin, Ehrich, & Manel, 2007). The percentage of polymorphic loci and genetic and epigenetic Shannon's information index was calculated separately per population using the MSAP_calc.r R script (Schulz et al., 2013). The methylation percentages were calculated using (Type II + Type III)/(Type I + Type II + Type III)*100%. And, the relative percentage of each type was calculated with (Type X)/(Type I + Type II + Type III + Type IV)*100% (Vergeer et al., 2012). The methylation percentages were calculated separately per environment (Field vs. Common garden), per country and for each population. They were subjected to analysis of variance (ANOVA) to test for significant differences. We calculated distance matrices both on individual and population level using GENALEX 6.5 (Peakall & Smouse, 2012). These matrices were imported in the R environment and used for further analysis. Principal coordinate analysis (PCoA) was performed and the principal coordinate values were plotted for AFLP, MS‐AFLP field and MS‐AFLP common garden using the individual‐level pairwise distance matrices using the pcoa() function from the package Ape (Paradis, Claude, & Strimmer, 2004).To analyze the genetic and epigenetic variation between countries, among populations within countries and within populations, we used the analysis of molecular variance (AMOVA) framework (Meirmans, 2006), using the amova() function from the package Pegas with 9,999 permutations (Paradis, 2010). ɸ_ST_ values for AFLP, MS‐AFLP Field and MS‐AFLP Common garden were calculated separated by country using the AMOVA framework (Meirmans, 2006). In addition, we tested for the homogeneity of variances between populations in the distance matrices for AFLP, MS‐AFLP field, and MS‐AFLP common garden using the betadisper() function from the Vegan R package, which is a multivariate analogue of the Levene's test for homogeneity of variance (Oksanen et al., [Link]; Preite et al., 2015).

Using the distance matrixes, we tested for correlations between AFLP, MS‐AFLP field, MS‐AFLP common garden, phenotype data, and geographical distance on population level with Mantel and partial Mantel tests, using the function mantel() from the Vegan package with 1,500 permutations (Oksanen et al., [Link]).

For the phenotypic data from the common garden Euclidian distance matrices were calculated both on population and on individual level, for all traits, with 76 individuals that were present in both AFLP and MS‐AFLP common garden data sets. Correlations between individual based distance matrices of AFLP, MS‐AFLP common garden and common garden traits were tested with Mantel tests, using mantel() from the Vegan package, with 1,500 permutations. Correlations were calculated at population level, as genetic variation was only determined in common garden‐grown plants.

## 3 RESULTS

### 3.1 Phenotypic differences in the field and the common garden

**Figure 1:**
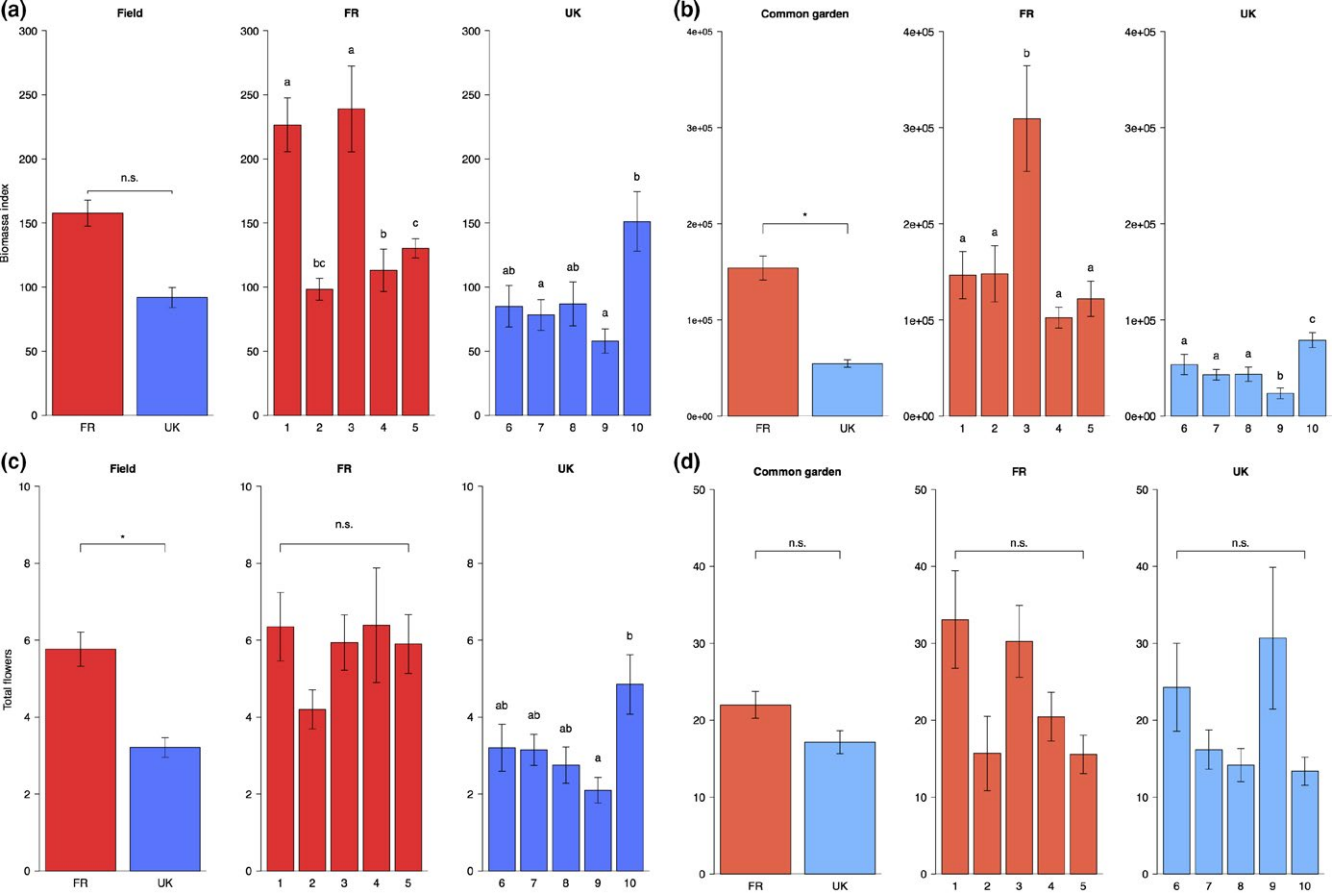
Biomass index (±SE) of plants grown in the field (a) and plants grown in the common garden (b) and total number of flowers (±SE) for plants grown in the field (c) and plants grown in the common garden (d). First, the differences between FR and the UK are shown, followed by the differences between FR and UK populations. Significant differences between countries are indicated with * (*p *< .05), significant differences between the populations per country were identified by post hoc comparisons and are indicated by lowercase letters

The selected populations showed large phenotypic differences, between and within countries. In general, plants from French populations grew larger and showed stronger flowering propensities compared to plants from British populations (Figure 1 and Appendix 7). When seedlings were grown in a common environment, most phenotypic differences remained, although less pronounced.

42.7% of the variance in biomass index in the French populations was explained by differences between populations, whereas in the UK, only 15.4% of the variation in biomass index was explained by differences among populations (Table 2 and Table S2 for statistics of field‐grown plants and common garden‐grown plants respectively). When plants were grown in a common environment, the variance in biomass index of French plants that was explained by the effect of population reduced significantly to 14.3%. In contrast, variances in biomass index that was explained by the different countries were similar in field and common garden situations (17.5% in the field versus 18.2% in the common garden; Table 2).

**Table 2:** The percentage of variance explained by country and population (separated by country) for both field and common garden phenotypic traits

Field				
Per country	Biomass index	Inflorescence height	No. of inflorescences	No. of flowers
Total variance	10,451	41.0	909	16.2
% country	17.5	0.00	**5.41**	**19.0**
FR				
Total variance	10,995	35.6	1.55	19.7
% population	**42.7**	0.00	0.00	0.00
UK				
Total variance	6,285	47.6	0.18	6.61
% population	**15.4**	**14.1**	1.75	**11.2**

Common Garden						
Per country	Biomass index (week 11)	Inflorescence height	No. of inflorescences	No. of flowers	Flowering time	Total biomass
Total variance	3.1534E+13	1,175	29.5	598	14.3	1,423
% country	**18.2**	5.74	0.09	0.47	**14.8**	**33.5**
FR						
Total variance	4.1473E+13	1,536	37.8	764	4.69	1,565
% population	**14.3**	**20.6**	**12.7**	2.43	0.00	**40.5**
UK						
Total variance	2.3479E+12	508	17.2	350	20.9	145
% population	**13.4**	0.00	4.05	0.08	0.00	**5.42**

Bold values indicate if the percentage of variance is significant (*p *< .05), based on ANOVAs of the linear mixed effect models (for country) and ANOVAs of the linear effect models (for FR and UK), for ANOVA tables see Table S2.

French plants produced significantly more flowers than British plants. These differences, however, were no longer significant when seedlings were grown in the common garden (Figure 1c,d and Table 2).

In the common garden, significant differences were observed in flowering time, with plants from UK populations flowering earlier than plants from FR populations. No significant differences were observed in bolting time and reproductive biomass between plants from FR and UK populations (Table 2). For inflorescence height, there were no significant differences between countries in the field‐grown plants, but in the common garden‐grown plants, FR populations had significantly taller inflorescences than UK populations (Figure S1).

In general, field‐grown plants showed stronger differentiation in traits than plants that were grown in a common environment. Plants from all populations, apart from population FR 3, became more similar when grown in the common garden. Population FR 3 strongly differentiated, both in the field and in the common garden, and was responsible for a considerable part of the variation between countries and populations. In both field and common garden plants, FR population 3 had the highest biomass (Figure 1), although this did not translate into increased flower production. For the UK populations, population 10 had the highest biomass and population 9 the lowest, in both field‐ and common garden‐grown plants.

The within‐population variances for the measured traits of all 10 populations were of the same order, and did not show significant differences between populations. Therefore we conclude that mother effects are likely to be minimal.

### 3.2 Genetic and epigenetic variation

Relatively high levels of genetic and epigenetic diversity were observed (Table 1). The mean percentage of polymorphic genetic bands was 74.8%, and the mean percentage of polymorphic epigenetic bands was 64.2% for the field‐grown plants and 69.4% for the common garden‐grown plants (Table 1). No private bands were observed. The average Shannon's index for the genetic diversity was 0.570, which is similar to the epigenetic diversity Shannon's indexes for both field‐grown and common garden‐grown plants (mean *H*_epi_ = 0.514 and 0.546, respectively).

**Figure 2:**
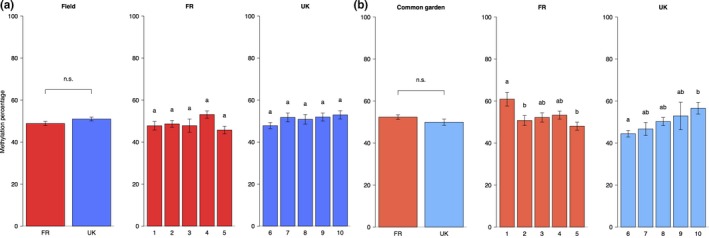
Methylation percentage (±SE) of plants grown in the field (a) and plants grown in the common garden (b). First, the differences between FR and the UK are shown, followed by the differences between FR and UK populations. There were no significant differences between countries, significant differences between the populations per country were identified by post hoc comparisons and are indicated by lowercase letters

Average methylation percentage differed between populations. For the French populations, average methylation was influenced by the environment, indicated by a significant environment × country interaction effect (Figure 2, Table S3). When the influence of the environment was tested separately for FR and UK populations, both FR and UK showed significant effects of populations on methylation percentage but no interaction between environment and population. However, FR populations showed a significant effect of environment on methylation percentage (Table S3).

**Figure 3:**
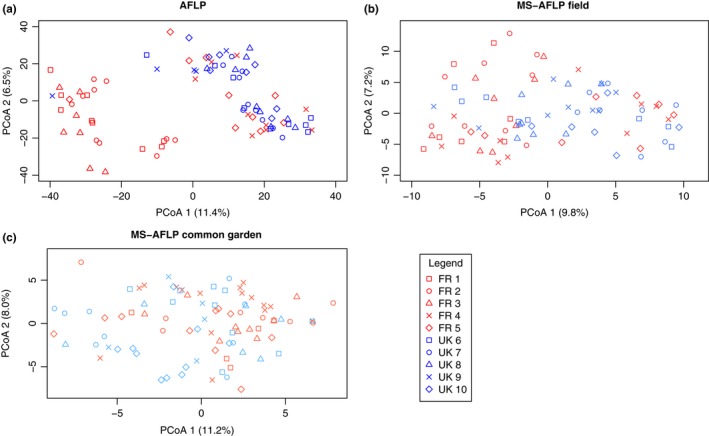
Principal coordinate analysis (PCoA) based on genetic (a, AFLP) and epigenetic distances from the field (b, MS‐AFLP Field) and the common garden (c, MS‐AFLP Common garden)

AMOVA tests showed that most genetic and epigenetic variation is explained between populations within countries rather than between countries (Table 3). This is reflected in the principal coordinate analysis (PCoA) based on the pairwise AFLP, MS‐AFLP field, and MS‐AFLP Common garden distance profiles (Figure 3). The most pronounced clustering in PCoA could be attributed to genetic differences (Table 3). The AFLP PCoA plot shows that the FR populations closest to the UK (populations FR 4 and FR 5) are genetically more similar to the UK populations (Figure 3a) than to the more Southern French populations. A comparable but less pronounced clustering was found in the epigenetic variation (MS‐AFLP Field PCoA; Figure 3b). In the MS‐AFLP Common garden plot, there is more within population variation than the AFLP and MS‐AFLP Field plots, but the molecular variance among populations was still significant (Figure 3c and Table 3). The variation partitioning among countries was in all three profiles relatively small, but was significant for all profiles (Table 3). The ɸ_ST_ of the AFLP and MS‐AFLP Field were comparable for both FR and UK populations, while it was smaller in the MS‐AFLP Common garden for both countries (Table 3). Only FR biomass index in the field showed a higher Q_ST_ than ɸ_ST_, all other traits in both environments had Q_ST_ values similar or smaller than ɸ_ST_ values (Table S4). Additionally, several traits showed very low among‐population variation and this differed between country and environment (Table S4).

**Table 3:** Variance partitioning (AMOVA) for AFLP, MS‐AFLP Field, and MS‐AFLP Common garden profiles. ɸ_ST_ was calculated separately per country. Bold values indicate *p*‐Value <.05

	AFLP							MS‐AFLP Field							MS‐AFLP Common garden						
	*df*	SSD	Mol. var. (%)	*p*‐Value	ɸ_ST_			*df*	SSD	Mol. var. (%)	*p*‐Value	ɸ_ST_			*df*	SSD	Mol. var. (%)	*p*‐Value	ɸ_ST_		
Among countries	1	9114	10.7	**.009**			*p*‐Value	1	202	4.10	**.036**			*p*‐Value	1	51.4	**1.49**	**.01**			*p*‐Value
Among populations within countries	8	23245	17.0	**<.0001**	FR	0.087	**<.0001**	8	857	13.1	**<.0001**	FR	0.082	**<.0001**	8	281	**8.03**	**.002**	FR	0.052	**.016**
					UK	0.066	**<.0001**					UK	0.063	**.004**					UK	0.053	**.038**
Within populations	78	74105	72.3					71	3334	82.8					78	1559	90.5				

*df*, degrees of freedom; SSD, sum of squared deviations.

Mol. var. (%): Molecular variance percentages were calculated from variance components sigma 2. *p*‐Values: derived from 9,999 permutations.

### 3.3 Genetic and epigenetic correlations

Population‐level genetic variation was positively correlated with the population‐level epigenetic variation in the field, and with the geographical distance between populations (Table 4). In contrast, when plants were grown in a common environment, no correlation between genetic and epigenetic variation was observed. Interestingly, epigenetic variation in the field and epigenetic variation in the common garden were not correlated.

**Table 4:** Outcome of population‐level Mantel tests correlations between AFLP, MS‐AFLP Field, MS‐AFLP common garden, and geographical distance of populations

	AFLP		MS‐AFLP Field		MS‐AFLP Common garden	
	*r*	*p*‐Value	*r*	*p*‐Value	*r*	*p*‐Value
AFLP						
MS‐AFLP Field	**.43**	**.006**				
MS‐AFLP Common garden	.06	.39	.22	.19		
Geographical distance	**.65**	**.004**	**.37**	**.01**	−.002	.47

Correlations and *p*‐Values were derived from 1,500 permutations. Bold values indicate a *p*‐Value <.05.

At the population level, both genetic variation and geographical distance showed significant correlations with biomass‐related traits, in both field‐ and common garden‐grown plants (Table 5). Geographic distance and biomass index in the field were, however, only marginally correlated. No significant correlations between epigenetic variation and phenotypic traits were observed, neither in the field nor in the common garden.

**Table 5:** Outcome of population‐level Mantel tests correlations between phenotypes and AFLP, MS‐AFLP Field, and MS‐AFLP common garden profiles and the geographical distance

Phenotype field	AFLP		MS‐AFLP Field		Geographical distance	
	*r*	*p*‐Value	*r*	*p*‐Value	*r*	*p*‐Value
Biomass Index	**.46**	**.04**	.13	.26	.22	.08
Inflorescence height	−.23	.88	.17	.27	−.12	.74
No. of inflorescences	−.11	.68	−.19	.79	−.04	.53
No. of flowers	.02	.35	−.06	.64	.05	.27

Phenotype common garden	AFLP		MS‐AFLP Common garden		Geographical distance	
	*r*	*p*‐Value	*r*	*p*‐Value	*r*	*p*‐Value
Biomass Index	**.48**	**.003**	−.24	.79	**.37**	**.01**
Inflorescence height	**.33**	**.04**	−.28	.81	.25	.06
No. of inflorescences	**.42**	**.02**	−.13	.72	.04	.35
No. of flowers	**.33**	**.04**	.04	.36	.003	.41
Flowering time	.03	.33	−.01	.62	.03	.41
Total biomass	**.57**	**.003**	−.27	.85	**.48**	**.002**

Field traits were only tested with MS‐AFLP data from field‐grown plants and common garden traits were only tested with MS‐AFLP data from common garden‐grown plants. Correlations and *p*‐Values were derived from 1,500 permutations. Bold values indicate a *p*‐Value <.05.

## 4 DISCUSSION

Here, we screened genetic and epigenetic variation in situ in natural *S. columbaria* populations. To test to what extent epigenetic differentiation was transmitted to a next generation, we did not only study field‐grown plants but also plants from the same population grown together in a common environment. Our study showed epigenetic population differentiation in both field and common garden environments, with a stronger differentiation in the field environment. This may indicate that part of the epigenetic variation was transmitted to a next generation (i.e., epigenetic memory), but also that a considerable part is induced by environment factors and may not be heritable. A clear correlation between genetic variation and epigenetic variation was observed in the natural field populations, whereas no correlation was found when seedlings were grown in a common environment. This suggests that the correlation between genetic and epigenetic variation in the field is based mainly on an environment‐induced component of epigenetic variation, where different field environments may induce population‐specific epigenetic patterns, or on random epimutations.

### 4.1 Genetic variation within and between populations

Our results show high genetic diversity within populations, which is in accordance with other studies on genetic diversity and variation of *S. columbaria* (Pluess & Stöcklin, 2004; Reisch & Poschlod, 2009; Waldmann & Andersson, 1998).

In our study, comparisons between Q_ST_ and ɸ_ST_ (for Q_ST_ and ɸ_ST_ see Table S4) revealed a higher Q_ST_ for biomass index in the field, suggesting that directional selection is likely involved (De Kort, Vandepitte, & Honnay, 2013; Merilä & Crnokrak, 2001). A considerable part of this higher Q_ST_ for biomass index was environmentally induced as was shown by the decreased Q_ST_ in the common garden‐grown plants. Additionally, all other traits in the field and common garden show either Q_ST_ comparable to ɸ_ST_ or a lower Q_ST_, which indicates that the differentiation can be explained by drift alone or that similar phenotypes are favored between populations (De Kort et al., 2013; Merilä & Crnokrak, 2001).

### 4.2 Epigenetic variation within and between populations

To date, several studies on epigenetic variation and differentiation have been performed that tested population differentiation in a common environment. These studies, however, showed mixed results, ranging from no epigenetic differentiation between populations (Avramidou et al., 2015; Nicotra et al., 2015) to differentiation only among geographically large regions (Preite et al., 2015), differentiation between habitats (Richards et al., 2012), or differentiation on both population and habitat level (Abratowska et al., 2012). Results of field studies of epigenetic population variation in natural field‐sampled plants varied between epigenetic differentiation between different habitats (Lira‐Medeiros et al., 2010; Rico et al., 2014) and epigenetic differentiation among populations habitat (Foust et al., 2016; Schulz et al., 2014). These field studies show that, when there are environmental differences, populations often show epigenetic differentiation. However, with field studies alone, it is not possible to show if the observed variation can mainly be attributed to environmentally induced methylation changes and if this variation is transferred to future generations. Using common garden studies, on the other hand, it is unclear if observed epigenetic differentiation between populations is caused by genetic variation or by the heritable component of environmentally induced methylation variation in field‐grown parental individuals. Taken together, the results from these studies emphasize the need to study epigenetic variation in both the natural field environment and in a common environment. Studies that screen for epigenetic variation in both field and common garden are still rare, while the combination of environments is necessary to draw stronger conclusions about adaptive epigenetic variation (Robertson & Richards, 2015a). Our experimental design compared epigenetic variation in the natural field environment and in a common garden. This design allowed us to expose the influence of natural field environments on epigenetic variation, as the key difference between the natural field data and the common garden data is the presence or absence of these population‐specific environmental differences.

While a large part of genetic and epigenetic variation was partitioned within populations, a significant part of the variation was partitioned between countries and between populations. In the common garden, the between‐population variance component was reduced compared to the field, which led to an increase in the within‐population variance component. However, there was still a significant differentiation between countries and populations and this fraction was comparable to the field. Our comparison of epigenetic differentiation between field and common garden‐grown plants showed two important results. First, part of the epigenetic differentiation remains intact in the common garden, and second, population differentiation in a common environment is smaller than in different natural (field) environments. This indicates that at least part of the epigenetic differentiation is transmitted to a next generation but also that a considerable part of the epigenetic differentiation is environmentally induced or a result of random epimutations and disappears when all plants are grown in a common environment.

### 4.3 Correlation between genetic and epigenetic variation

We determined genetic variation using common garden‐grown plants. Because these plants are the direct offspring of field‐grown plants, we assume genetic population patterns to be similar between field‐ and common garden‐grown plants. Epigenetic patterns may, however, differ between field‐ and common garden‐grown plants if epigenetic differences are environment‐induced or caused by random epimutations. Our results showed a significant correlation between genetic variation and epigenetic variation in field‐grown plants, but not between genetic variation and epigenetic variation in common garden‐grown plants. Moreover, epigenetic variation in the field‐grown plants was not correlated with epigenetic variation in the common garden‐grown plants. Also, when the genetic and epigenetic patterns of individual plants from the common garden were compared, no significant correlation between genetic and epigenetic variation was observed. A possible explanation is that epigenetic variation is largely environment induced which is not inherited to next generations. If natural environments induce different epigenetic profiles in different populations, such population‐specific induced epigenetic profiles may show a statistical association with genetic divergence between the populations. If this variation is not inherited, then this association will disappear. There still was significant epigenetic population differentiation in the common garden, which could in principle be caused by either heritable methylation variation (possibly genetically controlled) that is unsusceptible to the environment and/or environmentally induced methylation variation in the field that is heritable. Random epimutations could also contribute to these findings. Unfortunately, this study does not allows to distinguish underlying mechanism of these results.

### 4.4 Correlation between phenotype, genetic, and epigenetic variation

Phenotypic population differentiation was observed in the field and, although less pronounced, in the common garden. Population‐level phenotypic variation was correlated with genetic variation in both the field and in the common garden, and for the common garden when individual plants were compared. However, we found no correlations between traits and epigenetic variation, neither in the field nor in the common garden. This could imply that methylation variation does not affect phenotypic variation in Scabiosa columbaria. This would, however, contrast results of an earlier study on the same species in which a strong relation between DNA methylation and phenotypic variation was revealed (Vergeer et al., 2012). Moreover, it is widely accepted that epigenetic variation may significantly affect phenotypic variation (Bossdorf et al., 2010; Cortijo et al., 2014; Cubas et al., 1999; Johannes et al., 2009; Zhang, Fischer, Colot, & Bossdorf, 2013), although how exactly gene expression and phenotype are influenced by DNA methylation, it is not entirely understood and often no obvious connection between phenotype, gene expression and DNA methylation is observed (Robertson & Richards, 2015b; Schrey et al., 2013). In this study, AFLP and MS‐AFLP methods were used to analyze genetic and epigenetic variation. Although these methods have proven to be useful methods to analyze overall correlations between genetic, epigenetic and phenotypic relatedness, they are not suitable to uncover functionality or direct links between phenotypic and genetic or epigenetic variation (Robertson & Richards, 2015b; Schrey et al., 2013). In order to pinpoint the mechanic link between methylation and phenotype, other in‐depth methods such as next‐generation sequencing are necessary (van Gurp et al., 2016; Robertson & Richards, 2015b; Schrey et al., 2013).

## 5 CONCLUSIONS

Natural populations of *S. columbaria* showed substantial amounts of genetic and epigenetic variation with strong differentiation between countries and populations. By comparing field‐grown plants with seedlings that were grown in a common test environment, we showed that a considerable part of epigenetic differentiation is not heritable, and presumably environmentally induced. Only a small part is transmitted to the next generation, leading to epigenetic differentiation that is detectable also in the next‐generationcommon garden plants. This epigenetic memory can consist of heritable variation in methylation that is not sensitive to environments and possibly genetically based, environmentally induced variation that is heritable, or a combination of both. By comparing epigenetic variation in maternal plants in the field and a next generation that is grown in a common environment, our study provides useful insights into the environmental and genetic components of epigenetic variation.

## CONFLICT OF INTEREST

None declared.

## AUTHOR CONTRIBUTIONS

Conceived and designed the experiments: MG, PV, KV, and NO. Performed the experiments: MG and NW. Analyzed the data: MG and PV. Wrote the paper: MG, PV, KV, and NO.

## References

[ece33931-bib-0001] Abratowska, A. , Wąsowicz, P. , Bednarek, P. T. , Telka, J. , & Wierzbicka, M. (2012). Morphological and genetic distinctiveness of metallicolous and non‐metallicolous populations of *Armeria maritima* s.l. (Plumbaginaceae) in Poland. Plant Biology, 14, 586–595. https://doi.org/10.1111/j.1438-8677.2011.00536.x 22243547 10.1111/j.1438-8677.2011.00536.x

[ece33931-bib-0002] Alonso, C. , Pérez, R. , Bazaga, P. , Medrano, M. , & Herrera, C. M. (2016). MSAP markers and global cytosine methylation in plants: A literature survey and comparative analysis for a wild‐growing species. Molecular Ecology Resources, 16, 80–90. https://doi.org/10.1111/1755-0998.12426 25944158 10.1111/1755-0998.12426

[ece33931-bib-0003] Avramidou, E. V. , Ganopoulos, I. V. , Doulis, A. G. , Tsaftaris, A. S. , & Aravanopoulos, F. A. (2015). Beyond population genetics: Natural epigenetic variation in wild cherry (*Prunus avium*). Tree Genetics & Genomes, 11, 1–9.

[ece33931-bib-0004] Bates, D. , Maechler, M. , Bolker, B. , & Walker, S. (2014). lme4: Linear mixed‐effects models using Eigen and S4.

[ece33931-bib-0005] Bonin, A. , Ehrich, D. , & Manel, S. (2007). Statistical analysis of amplified fragment length polymorphism data: A toolbox for molecular ecologists and evolutionists. Molecular Ecology, 16, 3737–3758. https://doi.org/10.1111/j.1365-294X.2007.03435.x 17850542 10.1111/j.1365-294X.2007.03435.x

[ece33931-bib-0006] Bossdorf, O. , Arcuri, D. , Richards, C. L. , & Pigliucci, M. (2010). Experimental alteration of DNA methylation affects the phenotypic plasticity of ecologically relevant traits in *Arabidopsis thaliana* . Evolutionary Ecology, 24, 541–553. https://doi.org/10.1007/s10682-010-9372-7

[ece33931-bib-0007] Bossdorf, O. , Richards, C. L. , & Pigliucci, M. (2008). Epigenetics for ecologists. Ecology Letters, 11, 106–115. 18021243 10.1111/j.1461-0248.2007.01130.x

[ece33931-bib-0008] Cortijo, S. , Wardenaar, R. , Colomé‐Tatché, M. , Gilly, A. , Etcheverry, M. , Labadie, K. , … Roudier, F. (2014). Mapping the epigenetic basis of complex traits. Science, 343, 1145–1148. https://doi.org/10.1126/science.1248127 24505129 10.1126/science.1248127

[ece33931-bib-0009] Cubas, P. , Vincent, C. , & Coen, E. (1999). An epigenetic mutation responsible for natural variation in floral symmetry. Nature, 401, 157–161. https://doi.org/10.1038/43657 10490023 10.1038/43657

[ece33931-bib-0010] De Kort, H. , Vandepitte, K. , & Honnay, O. (2013). A meta‐analysis of the effects of plant traits and geographical scale on the magnitude of adaptive differentiation as measured by the difference between Q_ST_ and F_ST_ . Evolutionary Ecology, 27, 1081–1097. https://doi.org/10.1007/s10682-012-9624-9

[ece33931-bib-0011] Foust, C. , Preite, V. , Schrey, A. W. , Alvarez, M. , Robertson, M. , Verhoeven, K. , & Richards, C. (2016). Genetic and epigenetic differences associated with environmental gradients in replicate populations of two salt marsh perennials. Molecular Ecology, 25, 1639–1652. 26880043 10.1111/mec.13522

[ece33931-bib-0012] van Gurp, T. P. , Wagemaker, N. C. , Wouters, B. , Vergeer, P. , Ouborg, J. N. , & Verhoeven, K. J. (2016). epiGBS: Reference‐free reduced representation bisulfite sequencing. Nature Methods, 13, 322–324. 26855363 10.1038/nmeth.3763

[ece33931-bib-0013] Herrera, C. M. , & Bazaga, P. (2010). Epigenetic differentiation and relationship to adaptive genetic divergence in discrete populations of the violet Viola cazorlensis. New Phytologist, 187, 867–876. https://doi.org/10.1111/j.1469-8137.2010.03298.x 20497347 10.1111/j.1469-8137.2010.03298.x

[ece33931-bib-0014] Herrera, C. M. , Medrano, M. , & Bazaga, P. (2014). Variation in DNA methylation transmissibility, genetic heterogeneity and fecundity‐related traits in natural populations of the perennial herb *Helleborus foetidus* . Molecular Ecology, 23, 1085–1095. https://doi.org/10.1111/mec.12679 24471446 10.1111/mec.12679

[ece33931-bib-0015] Jablonka, E. , & Raz, G. (2009). Transgenerational epigenetic inheritance: Prevalence, mechanisms, and implications for the study of heredity and evolution. The Quarterly Review of Biology, 84, 131–176. https://doi.org/10.1086/598822 19606595 10.1086/598822

[ece33931-bib-0016] Johannes, F. , Porcher, E. , Teixeira, F. K. , Saliba‐Colombani, V. , Simon, M. , Agier, N. , … Audigier, P. (2009). Assessing the impact of transgenerational epigenetic variation on complex traits. PLoS Genetics, 5, e1000530. 19557164 10.1371/journal.pgen.1000530 PMC2696037

[ece33931-bib-0017] Keyte, A. L. , Percifield, R. , Liu, B. , & Wendel, J. F. (2006). Infraspecific DNA methylation polymorphism in cotton (*Gossypium hirsutum* L.). Journal of Heredity, 97, 444–450. https://doi.org/10.1093/jhered/esl023 16987937 10.1093/jhered/esl023

[ece33931-bib-0018] Kuznetsova, A. , Brockhoff, P. B. , & Christensen, R. H. B. (2015). lmerTest package: Tests in lenear mixed effects models. Journal of Statistical Software, 82, 1–26.

[ece33931-bib-0019] Lira‐Medeiros, C. F. , Parisod, C. , Fernandes, R. A. , Mata, C. S. , Cardoso, M. A. , & Ferreira, P. C. G. (2010). Epigenetic variation in mangrove plants occurring in contrasting natural environment. PLoS One, 5, e10326 https://doi.org/10.1371/journal.pone.0010326 20436669 10.1371/journal.pone.0010326 PMC2859934

[ece33931-bib-0020] Ma, K. F. , Song, Y. P. , Yang, X. H. , Zhang, Z. Y. , & Zhang, D. Q. (2013). Variation in genomic methylation in natural populations of Chinese white poplar. PLoS One, 8, e63977. 23704963 10.1371/journal.pone.0063977 PMC3660595

[ece33931-bib-0021] Medrano, M. , Herrera, C. M. , & Bazaga, P. (2014). Epigenetic variation predicts regional and local intraspecific functional diversity in a perennial herb. Molecular Ecology, 23, 4926–4938. https://doi.org/10.1111/mec.12911 25208110 10.1111/mec.12911

[ece33931-bib-0022] Meirmans, P. G. (2006). Using the AMOVA framework to estimate a standardized genetic differentiation measure. Evolution, 60, 2399–2402. https://doi.org/10.1111/j.0014-3820.2006.tb01874.x 17236430

[ece33931-bib-0023] Merilä, J. , & Crnokrak, P. (2001). Comparison of genetic differentiation at marker loci and quantitative traits. Journal of Evolutionary Biology, 14, 892–903. https://doi.org/10.1046/j.1420-9101.2001.00348.x

[ece33931-bib-0024] Nicotra, A. B. , Segal, D. L. , Hoyle, G. L. , Schrey, A. W. , Verhoeven, K. J. F. , & Richards, C. L. (2015). Adaptive plasticity and epigenetic variation in response to warming in an Alpine plant. Ecology and Evolution, 5, 634–647. https://doi.org/10.1002/ece3.1329 25691987 10.1002/ece3.1329 PMC4328768

[ece33931-bib-0025] Oksanen, J. , Guillaume Blanchet, F. , & Kindt, R. et al. (2011). vegan: Community Ecology Package. R package version 1.17‐9. Retrieved from: http://vegan.r-forge.r-project.org.

[ece33931-bib-0026] Ouborg, N. , Van Treuren, R. , & Van Damme, J. (1991). The significance of genetic erosion in the process of extinction. Oecologia, 86, 359–367. https://doi.org/10.1007/BF00317601 28312921 10.1007/BF00317601

[ece33931-bib-0027] Paradis, E. (2010). pegas: An R package for population genetics with an integrated–modular approach. Bioinformatics, 26, 419–420. https://doi.org/10.1093/bioinformatics/btp696 20080509 10.1093/bioinformatics/btp696

[ece33931-bib-0028] Paradis, E. , Claude, J. , & Strimmer, K. (2004). APE: Analyses of phylogenetics and evolution in R language. Bioinformatics, 20, 289–290. https://doi.org/10.1093/bioinformatics/btg412 14734327 10.1093/bioinformatics/btg412

[ece33931-bib-0029] Peakall, R. , & Smouse, P. E. (2012). GenAlEx 6.5: Genetic analysis in Excel. Population genetic software for teaching and research—an update. Bioinformatics, 28, 2537–2539. https://doi.org/10.1093/bioinformatics/bts460 22820204 10.1093/bioinformatics/bts460 PMC3463245

[ece33931-bib-0030] Picó, F. X. , Ouborg, N. J. , & Van Groenendael, J. M. (2004). Evaluation of the extent of among‐family variation in inbreeding depression in the perennial herb *Scabiosa columbaria* (Dipsacaceae). American Journal of Botany, 91, 1183–1189. https://doi.org/10.3732/ajb.91.8.1183 21653474 10.3732/ajb.91.8.1183

[ece33931-bib-0031] Pigliucci, M. (2005). Evolution of phenotypic plasticity: Where are we going now? Trends in Ecology & Evolution, 20, 481–486. https://doi.org/10.1016/j.tree.2005.06.001 16701424 10.1016/j.tree.2005.06.001

[ece33931-bib-0032] Pinheiro, J. , Bates, D. , DebRoy, S. , Sarkar, D. , & Team, R. C. (2015). nlme: Linear and nonlinear mixed effects models. R package version 3. 1–121.

[ece33931-bib-0033] Pluess, A. R. , & Stöcklin, J. (2004). Genetic diversity and fitness in *Scabiosa columbaria* in the Swiss Jura in relation to population size. Conservation Genetics, 5, 145–156. https://doi.org/10.1023/B:COGE.0000029999.10808.c2

[ece33931-bib-0034] Preite, V. , Snoek, L. , Oplaat, C. , Biere, A. , Van der Putten, W. , & Verhoeven, K. (2015). The epigenetic footprint of poleward range‐expanding plants in apomictic dandelions. Molecular Ecology, 24, 4406–4418. 26206253 10.1111/mec.13329

[ece33931-bib-0035] Rapp, R. A. , & Wendel, J. F. (2005). Epigenetics and plant evolution. New Phytologist, 168, 81–91. https://doi.org/10.1111/j.1469-8137.2005.01491.x 16159323 10.1111/j.1469-8137.2005.01491.x

[ece33931-bib-0036] Reisch, C. , & Poschlod, P. (2009). Land use affects flowering time: Seasonal and genetic differentiation in the grassland plant *Scabiosa columbaria* . Evolutionary Ecology, 23, 753–764. https://doi.org/10.1007/s10682-008-9270-4

[ece33931-bib-0037] Reyna‐Lopez, G. , Simpson, J. , & Ruiz‐Herrera, J. (1997). Differences in DNA methylation patterns are detectable during the dimorphic transition of fungi by amplification of restriction polymorphisms. Molecular and General Genetics MGG, 253, 703–710. 9079881 10.1007/s004380050374

[ece33931-bib-0038] Richards, C. L. , Schrey, A. W. , & Pigliucci, M. (2012). Invasion of diverse habitats by few Japanese knotweed genotypes is correlated with epigenetic differentiation. Ecology Letters, 15, 1016–1025. https://doi.org/10.1111/j.1461-0248.2012.01824.x 22731923 10.1111/j.1461-0248.2012.01824.x

[ece33931-bib-0039] Rico, L. , Ogaya, R. , Barbeta, A. , & Peñuelas, J. (2014). Changes in DNA methylation fingerprint of *Quercus ilex* trees in response to experimental field drought simulating projected climate change. Plant Biology, 16, 419–427. https://doi.org/10.1111/plb.12049 23889779 10.1111/plb.12049

[ece33931-bib-0040] Robertson, M. H. , & Richards, C. L. (2015a). Non‐genetic inheritance in evolutionary theory the importance of plant studies. Non‐Genetic Inheritance, 2, 3–11.

[ece33931-bib-0041] Robertson, M. , & Richards, C. (2015b). Opportunities and challenges of next‐generation sequencing applications in ecological epigenetics. Molecular Ecology, 24, 3799–3801. https://doi.org/10.1111/mec.13277 26197983 10.1111/mec.13277

[ece33931-bib-0042] Sáez‐Laguna, E. , Guevara, M.‐Á. , Díaz, L.‐M. , Sánchez‐Gómez, D. , Collada, C. , Aranda, I. , & Cervera, M.‐T. (2014). Epigenetic variability in the genetically uniform forest tree species *Pinus pinea* L. PLoS One, 9, e103145 https://doi.org/10.1371/journal.pone.0103145 25084460 10.1371/journal.pone.0103145 PMC4118849

[ece33931-bib-0043] Scheepens, J. , Stöcklin, J. , & Pluess, A. R. (2010). Unifying selection acts on competitive ability and relative growth rate in *Scabiosa columbaria* . Basic and Applied Ecology, 11, 612–618. https://doi.org/10.1016/j.baae.2010.08.008

[ece33931-bib-0044] Schrey, A. W. , Alvarez, M. , Foust, C. M. , Kilvitis, H. J. , Lee, J. D. , Liebl, A. L. , … Robertson, M. (2013). Ecological epigenetics: Beyond MS‐AFLP. Integrative and Comparative Biology, 53, 340–350. https://doi.org/10.1093/icb/ict012 23583961 10.1093/icb/ict012

[ece33931-bib-0045] Schulz, B. , Eckstein, R. L. , & Durka, W. (2013). Scoring and analysis of methylation‐sensitive amplification polymorphisms for epigenetic population studies. Molecular Ecology Resources, 13, 642–653. https://doi.org/10.1111/1755-0998.12100 23617735 10.1111/1755-0998.12100

[ece33931-bib-0046] Schulz, B. , Eckstein, R. L. , & Durka, W. (2014). Epigenetic variation reflects dynamic habitat conditions in a rare floodplain herb. Molecular Ecology, 23, 3523–3537. https://doi.org/10.1111/mec.12835 24943730 10.1111/mec.12835

[ece33931-bib-0047] Spitze, K. (1993). Population structure in *Daphnia obtusa*: Quantitative genetic and allozymic variation. Genetics, 135, 367–374. 8244001 10.1093/genetics/135.2.367 PMC1205642

[ece33931-bib-0048] Steinger, T. , Haldimann, P. , Leiss, K. , & Müller‐Schärer, H. (2002). Does natural selection promote population divergence? A comparative analysis of population structure using amplified fragment length polymorphism markers and quantitative traits. Molecular Ecology, 11, 2583–2590. https://doi.org/10.1046/j.1365-294X.2002.01653.x 12453241 10.1046/j.1365-294x.2002.01653.x

[ece33931-bib-0049] Sultan, S. E. (2000). Phenotypic plasticity for plant development, function and life history. Trends in Plant Science, 5, 537–542. https://doi.org/10.1016/S1360-1385(00)01797-0 11120476 10.1016/s1360-1385(00)01797-0

[ece33931-bib-0050] Van Treuren, R. , Bijlsma, R. , Ouborg, N. , & Van Delden, W. (1993). The significance of genetic erosion in the process of extinction. IV. Inbreeding depression and heterosis effects caused by selfing and outcrossing in *Scabiosa columbaria* . Evolution, 47, 1669–1680.https://doi.org/10.1111/j.1558-5646.1993.tb01259.x 28567996 10.1111/j.1558-5646.1993.tb01259.x

[ece33931-bib-0051] Vergeer, P. , Wagemaker, C. A. M. , & Ouborg, N. J. (2012). Evidence for an epigenetic role in inbreeding depression. Biology Letters, 8, 798–801. https://doi.org/10.1098/rsbl.2012.0494 22791708 10.1098/rsbl.2012.0494 PMC3441007

[ece33931-bib-0052] Verhoeven, K. J. F. , Jansen, J. J. , van Dijk, P. J. , & Biere, A. (2010). Stress‐induced DNA methylation changes and their heritability in asexual dandelions. New Phytologist, 185, 1108–1118. https://doi.org/10.1111/j.1469-8137.2009.03121.x 20003072 10.1111/j.1469-8137.2009.03121.x

[ece33931-bib-0053] Vos, P. , Hogers, R. , Bleeker, M. , Reijans, M. , Van de Lee, T. , Hornes, M. , … Kuiper, M. (1995). AFLP: A new technique for DNA fingerprinting. Nucleic Acids Research, 23, 4407–4414. https://doi.org/10.1093/nar/23.21.4407 7501463 10.1093/nar/23.21.4407 PMC307397

[ece33931-bib-0054] Waldmann, P. , & Andersson, S. (1998). Comparison of quantitative genetic variation and allozyme diversity within and between populations of Scabiosa canescens and S. columbaria. Heredity, 81, 79–86. https://doi.org/10.1046/j.1365-2540.1998.00379.x

[ece33931-bib-0055] Whitlock, M. C. (2008). Evolutionary inference from Q_ST_ . Molecular Ecology, 17, 1885–1896. https://doi.org/10.1111/j.1365-294X.2008.03712.x 18363667 10.1111/j.1365-294X.2008.03712.x

[ece33931-bib-0056] Wu, W.‐Q. , Yi, M. R. , Wang, X.‐F. , Ma, L.‐L. , Jiang, L. , Li, X.‐W. , … Liu, B. (2013). Genetic and epigenetic differentiation between natural *Betula ermanii* (Betulaceae) populations inhabiting contrasting habitats. Tree Genetics & Genomes, 9, 1321–1328. https://doi.org/10.1007/s11295-013-0641-9

[ece33931-bib-0057] Yu, Y. , Yang, X. , Wang, H. , Shi, F. , Liu, Y. , Liu, J. , … Liu, B. (2013). Cytosine methylation alteration in natural populations of *Leymus chinensis* induced by multiple abiotic stresses. PLoS One, 8, e55772 https://doi.org/10.1371/journal.pone.0055772 23418457 10.1371/journal.pone.0055772 PMC3572093

[ece33931-bib-0058] Zhang, Y. Y. , Fischer, M. , Colot, V. , & Bossdorf, O. (2013). Epigenetic variation creates potential for evolution of plant phenotypic plasticity. New Phytologist, 197, 314–322. https://doi.org/10.1111/nph.12010 23121242 10.1111/nph.12010

