# Supplementary material for: Epigenetic population differentiation in field‐ and common garden‐grown Scabiosa columbaria plants
Source: Ecol Evol. 2018 Feb 25;8(6):3505–17. doi: 10.1002/ece3.3931 (PMC5869358; doi:10.1002/ece3.3931)
Supplement: Supplementary file 1 [file ECE3-8-3505-s001.docx]

Table S1. Primers used for AFLP and MS-AFLP.

| AFLP | | MS-AFLP | |
| --- | --- | --- | --- |
| EcoRI-Adapter I | ctcgtagactgcgtacc | EcoRI-Adapter I | ctcgtagactgcgtacc |
| EcoRI-Adapter II | aattggtacgcagtctac | EcoRI-Adapter II | aattggtacgcagtc |
| MseI- Adapter I | gacgatgagtcctgag | HM-Adapter I | gatcatgagtcctgct |
| MseI- Adapter II | tactcaggactcat | HM-Adapter II | cgagcaggactcatga |
| MseI-T | gatgagtcctgagtaaT | HM-T | atcatgagtcctgctcggt |
| EcoRI-A | gactgcgtaccaattca | EcoRI-A | gactgcgtaccaattca |

**Table S2.** ANOVA table of the linear mixed-effects model (for country) and linear models (for FR and UK) of the Field and Common garden grown plants. Bold values indicate if the percentage of variance is significant (*p*-value < 0.05).

|  |  | Field | | | | | Common garden | | | | |
| --- | --- | --- | --- | --- | --- | --- | --- | --- | --- | --- | --- |
|  |  | Country |  | FR |  | UK | Country |  | FR |  | UK |
|  | numDF | 1 |  | 4 |  | 4 | 1 |  | 4 |  | 4 |
| Biomass index | denDF | 79.5 |  | 95 |  | 95 | 8.39 |  | 231 |  | 143 |
|  | F-value | 4.28 |  | 13.9 |  | 3.69 | 8.68 |  | 3.88 |  | 8.66 |
|  | *p*-value | 0.07 |  | <0.0001 |  | 0.008 | 0.02 |  | 0.005 |  | <0.0001 |
| Inflorescence height | denDF | 79.8 |  | 95 |  | 95 | 8.21 |  | 228 |  | 144 |
|  | F-value | 0.04 |  | 0.44 |  | 3.21 | 3.04 |  | 5.69 |  | 1.03 |
|  | *p*-value | 0.856 |  | 0.781 |  | 0.016 | 0.119 |  | 0.0002 |  | 0.395 |
| # Inflorescences | denDF | 198 |  | 95 |  | 95 | 7.11 |  | 99 |  | 66 |
|  | F-value | 6.72 |  | 1.21 |  | 1.10 | 1.25 |  | 3.14 |  | 2.01 |
|  | *p*-value | 0.010 |  | 0.310 |  | 0.363 | 0.300 |  | 0.018 |  | 0.103 |
| # Flowers | denDF | N.A. |  | N.A. |  | N.A. | 6.06 |  | 99 |  | 66 |
|  | F-value | N.A. |  | N.A. |  | N.A. | 1.31 |  | 1.57 |  | 0.68 |
|  | *p*-value | N.A. |  | N.A. |  | N.A. | 0.296 |  | 0.187 |  | 0.608 |
| Bolting time | denDF | N.A. |  | N.A. |  | N.A. | 6.67 |  | 127 |  | 86 |
|  | F-value | N.A. |  | N.A. |  | N.A. | 1.85 |  | 1.90 |  | 0.29 |
|  | *p*-value | N.A. |  | N.A. |  | N.A. | 0.218 |  | 0.114 |  | 0.881 |
| Flowering time | denDF | N.A. |  | N.A. |  | N.A. | 4.42 |  | 88 |  | 74 |
|  | F-value | N.A. |  | N.A. |  | N.A. | 16.4 |  | 0.37 |  | 1.52 |
|  | *p*-value | N.A. |  | N.A. |  | N.A. | 0.013 |  | 0.829 |  | 0.205 |
| Total biomass | denDF | N.A. |  | N.A. |  | N.A. | 8.21 |  | 227 |  | 143 |
|  | F-value | N.A. |  | N.A. |  | N.A. | 8.24 |  | 15.2 |  | 3.48 |
|  | *p*-value | N.A. |  | N.A. |  | N.A. | 0.020 |  | <0.0001 |  | 0.010 |

numDF is the number of treatment groups and denDF is the number of replicates per group.

Table S3. Results of the ANOVA table for the number of bands, methylation percentage and the percentages per type. See Table S4 for the differences between countries and between populations within country. Significant *p*-values are indicated in bold.

|  | Total number of bands | | |  | Methylation percentage | | |  | Type I percentage | | |  | Type II percentage | | |  | Type III percentage | | |  | Type IV percentage | | |
| --- | --- | --- | --- | --- | --- | --- | --- | --- | --- | --- | --- | --- | --- | --- | --- | --- | --- | --- | --- | --- | --- | --- | --- |
| Per country |  |  |  |  |  |  |  |  |  |  |  |  |  |  |  |  |  |  |  |  |  |  |  |
|  | Df | F-value | *p*-value |  | Df | F-value | *p*-value |  | Df | F-value | *p*-value |  | Df | F-value | *p*-value |  | Df | F-value | *p*-value |  | Df | F-value | *p*-value |
| Environment | 1 | **21.9** | **<0.001** |  | 1 | 1.19 | 0.277 |  | 1 | **21.9** | **<0.001** |  | 1 | 0.63 | 0.429 |  | 1 | **7.08** | **0.009** |  | 1 | **14.2** | **<0.001** |
| Country | 1 | 0.62 | 0.456 |  | 1 | 0.00 | 0.997 |  | 1 | 0.61 | 0.458 |  | 1 | 0.05 | 0.824 |  | 1 | 0.10 | 0.759 |  | 1 | 0.14 | 0.721 |
| Environment * country | 1 | **13.9** | **<0.001** |  | 1 | **4.47** | **0.04** |  | 1 | **13.9** | **<0.001** |  | 1 | **7.64** | **0.006** |  | 1 | **5.11** | **0.025** |  | 1 | 0.05 | 0.819 |
|  |  |  |  |  |  |  |  |  |  |  |  |  |  |  |  |  |  |  |  |  |  |  |  |
| FR |  |  |  |  |  |  |  |  |  |  |  |  |  |  |  |  |  |  |  |  |  |  |  |
| Environment | 1 | 0.48 | 0.492 |  | 1 | **6.39** | **0.013** |  | 1 | **6.99** | **0.010** |  | 1 | **7.41** | **0.008** |  | 1 | 0.02 | 0.885 |  | 1 | 0.48 | 0.491 |
| Population | 4 | 0.45 | 0.776 |  | 4 | **2.93** | **0.026** |  | 4 | 1.54 | 0.198 |  | 4 | **3.95** | **0.006** |  | 4 | 2.09 | 0.090 |  | 4 | 0.43 | 0.784 |
| Environment * population | 4 | 0.85 | 0.497 |  | 4 | 2.06 | 0.094 |  | 4 | 2.21 | 0.076 |  | 4 | 1.36 | 0.254 |  | 4 | 1.65 | 0.169 |  | 4 | 0.87 | 0.488 |
|  |  |  |  |  |  |  |  |  |  |  |  |  |  |  |  |  |  |  |  |  |  |  |  |
| UK |  |  |  |  |  |  |  |  |  |  |  |  |  |  |  |  |  |  |  |  |  |  |  |
| Environment | 1 | **31.3** | **<0.001** |  | 1 | 0.43 | 0.516 |  | 1 | **7.39** | **0.008** |  | 1 | 1.29 | 0.261 |  | 1 | **11.1** | **0.001** |  | 1 | **31.3** | **<0.001** |
| Population | 4 | **2.59** | **0.044** |  | 4 | **3.88** | **0.007** |  | 4 | 1.00 | 0.416 |  | 4 | **3.34** | **0.015** |  | 4 | **2.77** | **0.034** |  | 4 | 2.59 | 0.044 |
| Environment * population | 4 | 0.57 | 0.688 |  | 4 | 0.90 | 0.472 |  | 4 | 0.26 | 0.900 |  | 4 | 0.52 | 0.719 |  | 4 | 1.35 | 0.262 |  | 4 | 0.56 | 0.690 |

Table S4. Qst values of all shared traits between Field and Common garden grown plants. For comparisons ɸst values from Table 3 were included.

|  | Biomass Index |  | Inflorescence height |  | # Inflorescences |  | # Flowers |  | ɸst | ɸst AFLP |  | ɸst MS-AFLP Field |  | ɸst MS-AFLP Common garden |
| --- | --- | --- | --- | --- | --- | --- | --- | --- | --- | --- | --- | --- | --- | --- |
| Field |  |  |  |  |  |  |  |  |  |  |  |  |  |  |
| FR | 0.2715 |  | 0.0000 |  | 0.0000 |  | 0.0000 |  | FR | 0.087 |  | 0.082 |  | 0.052 |
| UK | 0.0831 |  | 0.0758 |  | 0.0088 |  | 0.0593 |  | UK | 0.066 |  | 0.063 |  | 0.053 |
| Common garden |  |  |  |  |  |  |  |  |  |  |  |  |  |  |
| FR | 0.0768 |  | 0.1147 |  | 0.0677 |  | 0.0123 |  |  |  |  |  |  |  |
| UK | 0.0716 |  | 0.0000 |  | 0.0207 |  | 0.0004 |  |  |  |  |  |  |  |

Table S5. Outcome of partial Mantel tests per population. Shown are the correlation between phenotype and AFLP (corrected with MS-AFLP Field or MS-AFLP Common garden), and both MS-AFLP profiles (corrected with AFLP). Field traits were only tested with MS-AFLP Field and common garden traits were only tested with MS-AFLP Common garden. Correlations and *p*-values were derived from 1500 permutations. Bold values indicate a *p*-value < 0.05.

|  | AFLP | |  | MS-AFLP Field | |
| --- | --- | --- | --- | --- | --- |
| Phenotype field | r | *p-value* |  | r | *p-value* |
| Biomass Index | **0.46** | **0.027** |  | -0.09 | 0.653 |
| Inflorescence height | -0.34 | 0.994 |  | 0.30 | 0.137 |
| # Inflorescences | -0.03 | 0.468 |  | -0.15 | 0.736 |
| # Flowers | 0.05 | 0.258 |  | -0.07 | 0.690 |
| # Flowers per inflorescence | 0.14 | 0.150 |  | -0.03 | 0.552 |
|  | AFLP | |  | MS-AFLP Common garden | |
| Phenotype common garden | r | *p-value* |  | r | *p-value* |
| Biomass Index | **0.51** | **0.001** |  | -0.30 | 0.897 |
| Inflorescence height | **0.36** | **0.027** |  | -0.32 | 0.912 |
| # Inflorescences | **0.43** | **0.019** |  | -0.16 | 0.809 |
| # Flowers | **0.32** | **0.045** |  | 0.03 | 0.420 |
| Bolting time | -0.14 | 0.805 |  | -0.25 | 0.904 |
| Flowering time | 0.03 | 0.348 |  | -0.10 | 0.638 |
| Reproductive biomass | **0.47** | **0.003** |  | -0.29 | 0.883 |
| Non-reproductive biomass | **0.71** | **0.003** |  | -0.39 | 0.991 |
| Total biomass | **0.61** | **0.002** |  | -0.36 | 0.973 |

Table S6. Mantel tests based on individual AFLP. MS-AFLP Common garden and phenotypic traits from the common garden distance matrixes. Correlations and *p*-values were derived from 1500 permutations. Bold values indicate a *p*-value < 0.05.

|  | AFLP | |  | MS-AFLP Common garden | |
| --- | --- | --- | --- | --- | --- |
|  | r | *p*-value |  | r | *p*-value |
| MS-AFLP Common garden | -0.06 | 0.80 |  |  |  |
| Biomass Index | **0.14** | **0.03** |  | -0.03 | 0.64 |
| Inflorescence height | 0.04 | 0.26 |  | -0.02 | 0.57 |
| # Inflorescences | 0.07 | 0.13 |  | -0.03 | 0.69 |
| # Flowers | 0.01 | 0.38 |  | -0.04 | 0.69 |
| Bolting time | 0.04 | 0.14 |  | -0.04 | 0.83 |
| Flowering time | -0.01 | 0.62 |  | 0.01 | 0.27 |
| Total biomass | **0.12** | **0.05** |  | -0.03 | 0.64 |

**
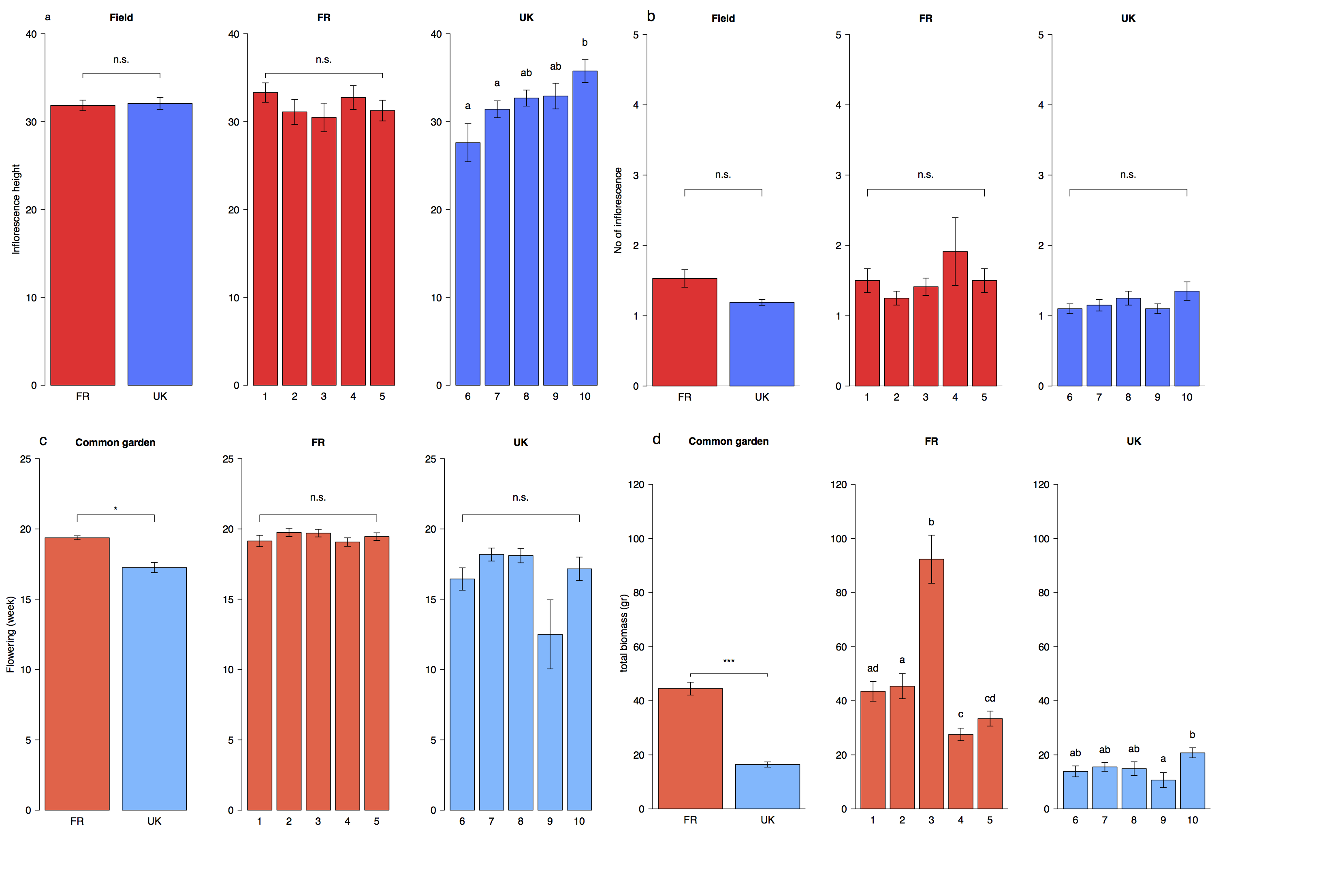
*Figure S1:***

**
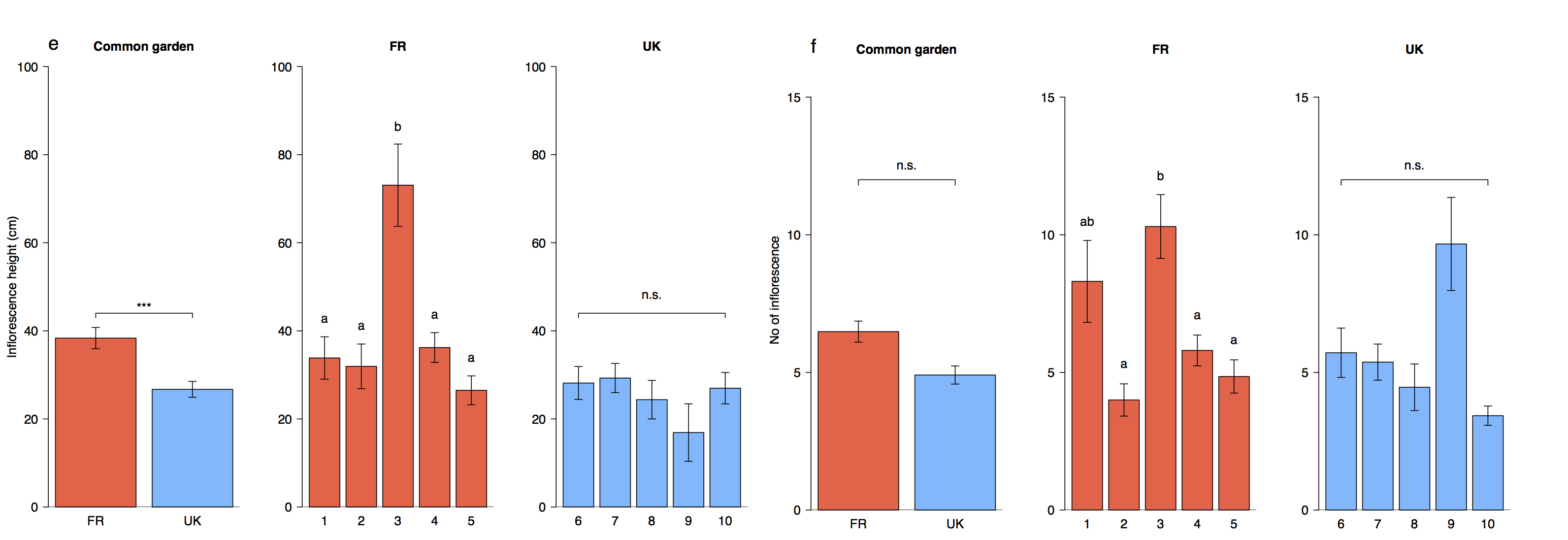
**

**Figure. S1.** Differences between FR and UK field (a and b) and common garden (c till f) grown plants (± SE) for inflorescence height (a), number of inflorescences (b) flowering time (c), total biomass (d), inflorescence height (e) and number of inflorescences (f). First the differences between FR and the UK are shown, significant differences are indicated with * (*p*-value <0.05) or n.s. when they are not significantly different. Than the differences between FR and UK populations is shown, with the significant differences between the populations per country were identified by post hoc comparisons and are indicated by lowercase letters.
